# The pseudogene derived from long non-coding RNA DUXAP10 promotes colorectal cancer cell growth through epigenetically silencing of p21 and PTEN

**DOI:** 10.1038/s41598-017-07954-7

**Published:** 2017-08-04

**Authors:** Yifan Lian, Yetao Xu, Chuanxing Xiao, Rui Xia, Huangbo Gong, Peng Yang, Tao Chen, Dongdong Wu, Zeling Cai, Jianping Zhang, Keming Wang

**Affiliations:** 1 Department of Gastroenterology, Zhongshan Hospital affiliated to Xiamen University, Xiamen, 361004 Fujian People’s Republic of China; 2 Department of Oncology, Second Affiliated Hospital, Nanjing Medical University, Nanjing, 210000 Jiangsu People’s Republic of China; 3 Department of Obstetrics and Gynecology, the First Affiliated Hospital of Nanjing Medical University, Nanjing, 210000 Jiangsu People’s Republic of China; 4 Department of Laboratory, Nanjing Chest Hospital, Nanjing, 210029 Jiangsu People’s Republic of China; 5 Department of General Surgery, Second Affiliated Hospital, Nanjing Medical University, Nanjing, 210000 Jiangsu People’s Republic of China

## Abstract

Recently, substantial evidence has demonstrated that pseudogene derived lncRNAs are crucial regulators of cancer development and progression. DUXAP10,a pseudogene derived long non-coding RNA(lncRNA), is overexpression in colorectal cancer (CRC), but its expression pattern, biological function and underlying mechanism in CRC is still undetermined. In this study, we observed that DUXAP10 was up-regulated in CRC tissues which was positively correlated with advanced pathological stages, larger tumor sizes and lymph node metastasis. Additionally, knockdown of DUXAP10 inhibited cell proliferation, induced cell apoptosis and increase the number of G0/G1 cells significantly in the HCT116 and SW480 cell lines. Moreover, DUXAP10 silencing inhibited tumor growth *in vivo*. Further mechanism study showed that, by binding to histone demethylase lysine-specific demethylase 1 (LSD1), DUXAP10 promote CRC cell growth and reduced cell apoptosis through silencing the expression of p21 and phosphatase and tensin homolog (PTEN) tumor suppressor. Our findings suggested that the pseudogene-derived from lncRNA DUXAP10 promotes the biological progression of CRC and is likely to be a potential therapeutic target for CRC intervention.

## Introduction

Colorectal carcinoma (CRC) is a common type of cancer in American and China, thus representing a major public health problem^1, 2^. It accounts for over 9% of all malignancy incidence, with estimated 1.4 million cases occurring in 2012 worldwide^3, 4^. Moreover, the global burden is expected to further increase due to the growth and aging of the population and the adoption of westernized behaviors and lifestyle^3^. Colorectal carcinogenesis is a complicated biological process that results from the dysregulation of many cancer-related genes. Therefore, a greater understanding of the molecular mechanisms involved in the development and progression of colorectal cancer is essential to develop targeted strategies that could alleviate the burden of the disease.

In the past decade, the significance of nonprotein-coding functional elements in the human genome has emerged from the water and been identified as an important revelation in post-genomic biology^5–7^. Recently, benefiting from the improvement of bioinformatics methods and large scale sequencing technique, tens of thousands of pseudogenes as well as numerous long non-coding RNA (lncRNA) genes were identified^8, 9^. Briefly, pseudogenes are the results of duplicated genes, which have lost their protein-coding capacity through molecular events such as point or frameshift mutations^8^. Interestingly, it is becoming apparent that many pseudogenes are transcribed into long non-coding RNAs, with proven biological functions^10^. They play a plethora of roles at multiple levels (DNA, RNA or protein) in diverse pathological processes, especially in carcinogenesis^11, 12^. In the recent reported study by Ma H and colleagues, the pseudogene derived from long non-coding RNA DUXAP8 can promote gastric cancer cell proliferation and tumorigenesis through epigenetically silencing PLEKHO1 transcription^13^. In addition, the pseudogene-expressed lncRNA RSU1P2 was found to be significantly up-regulated in cervical cancer, and functioned as an oncogene in cervical cancer cells^14^. Therefore, pseudogene-expressed lncRNAs have been highlighted as key factors in cancer research.

DUXAP10 is previously found to be overexpressed in non small cell lung cancer (NSCLC) and promotes NSCLC cells proliferation. However, the biological functions and underline mechanism of DUXAP10 in the control of CRC tumorigenes is have not been documented. These prompted us to explore the role of DUXAP10 in human CRC^15^. In the present study, we investigated that pseudogene-expressed lncRNA DUXAP10 was aberrantly expressed in CRC and positively associated with tumor size, pathological stage and lymphatic metastasis. Furthermore, functional analysis revealed that DUXAP10 could promote CRC cell growth both *in vitro* and *in vivo*. Mechanism studies indicated that DUXAP10 is an oncogenic pseudogene-expressed lncRNA that promotes tumorigenesis through epigenetically silencing p21 and PTEN expression.

## Results

### DUXAP10 is upregulated in human CRC tissues and is positively correlated with larger tumor size, advanced TNM stage and lymph node metastasis

**Figure 1:**
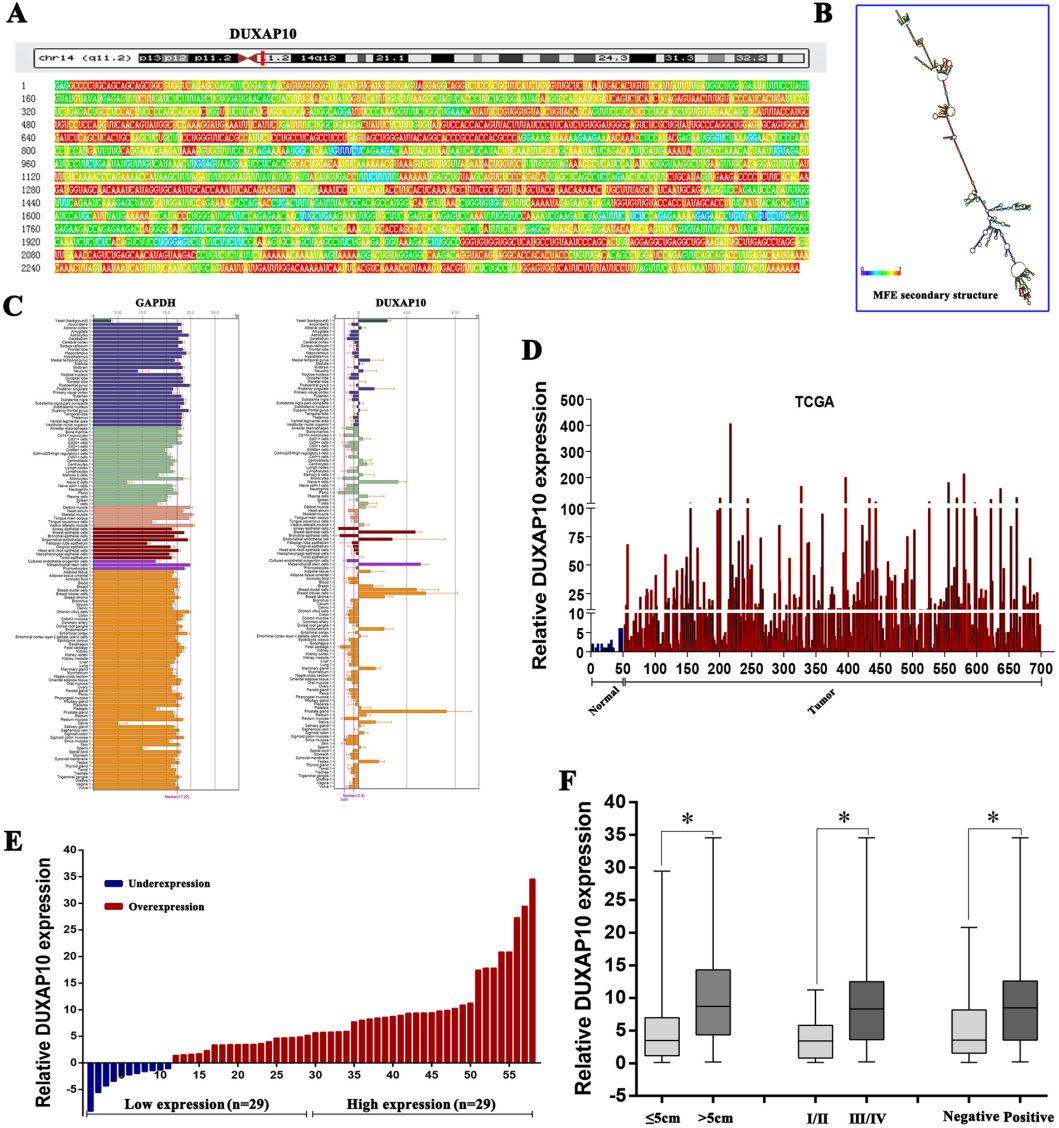
Relative DUXAP10 expression in CRC tissues and its clinical significance. (**A**) The full sequence of DUXAP10 is published in the NCBI database (NR_110526.1). (**B**) Prediction of DUXAP10 structure based on minimum free energy (MFE) and partition function. Color scale indicates the confidence for the prediction for each base with shades of red indicating strong confidence. (http://rna.tbi.univie.ac.at/). (**C**) Low DUXAP10 expression levels in normal human colorectal tissues. The protein coding gene GAPDH was used as a control. (**D**) Relative expression of DUXAP10 in colorectal cancer tissues compared with normal tissue was analyzed by using TCGA data. (**E**) Relative expression of DUXAP10 in CRC tissues compared with corresponding adjacent normal tissues (n = 58), and DUXAP10 expression was classifid into two groups. **(F)** DUXAP10 expression was significantly higher in patients with a larger tumor size, a higher pathological stage and lymph node metastasis (shown as ΔCT). Bars: s.d., *P < 0.05, **P < 0.01.

The DUXAP10 gene is located at the chromosomal locus 14q11.2 and encodes a 2398 bp transcript (Fig. 1A; https://www.ncbi.nlm.nih.gov/nuccore/NR_110526.1). In addition, BioGPS dataset analysis indicated that the DUXAP10 gene is not expressed in normal human colorectal tissues (A z-score > 5 suggests that a gene is expressed in a particular tissue) (Fig. 1C). Moreover, analysis of TCGA colorectal cancer and normal tissues RNA sequencing data showed that DUXAP10 expression is upregulated in tumor tissues compared with normal tissues (Fig. 1D). To validate the analysis finding, we detected DUXAP10 expression in an cohort of 58 pair colorectal cancer and normal tissues using qPCR. The results confirmed that DUXAP10 expression is increased in CRC tissues compared with matched non-tumor tissues (Fig. 1E). To further investigated the relationship between DUXAP10 expression and clinical pathological features, we divided the samples into high (above the median, n = 29) and low (below the median, n = 29) DUXAP10 expression groups according to the median value of it levels (Fig. 1E). A chi-square test was then performed to evaluate the clinicopathological features between the two groups. As shown in Table 1, increased DUXAP10 in CRC tissues were significantly correlated with larger tumor sizes (P = 0.033), advanced TNM stages (P = 0.016) and lymph node metastasis (P = 0.017) (Fig. 1F,G). However, several other clinical parameters were found not to be significantly associated with DUXAP10 expression (Table 1). Therefore, we speculate that DUXAP10 expression may correlate with the malignant behaviors of colorectal carcinoma.

**Table 1:** Correlation between DUXAP10 expression and clinicopathologic characteristics of patients with CRC.(n = 58).

Characteristics	DUXAP10		*P*
	Low No. Cases	High No. Cases	Chi-squared test, *P*-value
**Age(years)**	(n = 29)	(n = 29)	0.777
**≤**60	10	8	
>60	19	21	
**Gender**			0.596
Male	15	18	
Female	14	11	
**Tumor size(cm)**			0.033
**≤**5	21	12	
>5	8	17	
**TNM Stage**			0.016
I/II	17	7	
III/IV	12	22	
**Lymph node metastasis**			0.017
Positive	8	18	
Negative	21	11	
**Distant metastasis**		0.179	
Positive	3	8	
Negative	26	21	

### Modulation of DUXAP10 expression in CRC cells

**Figure 2:**
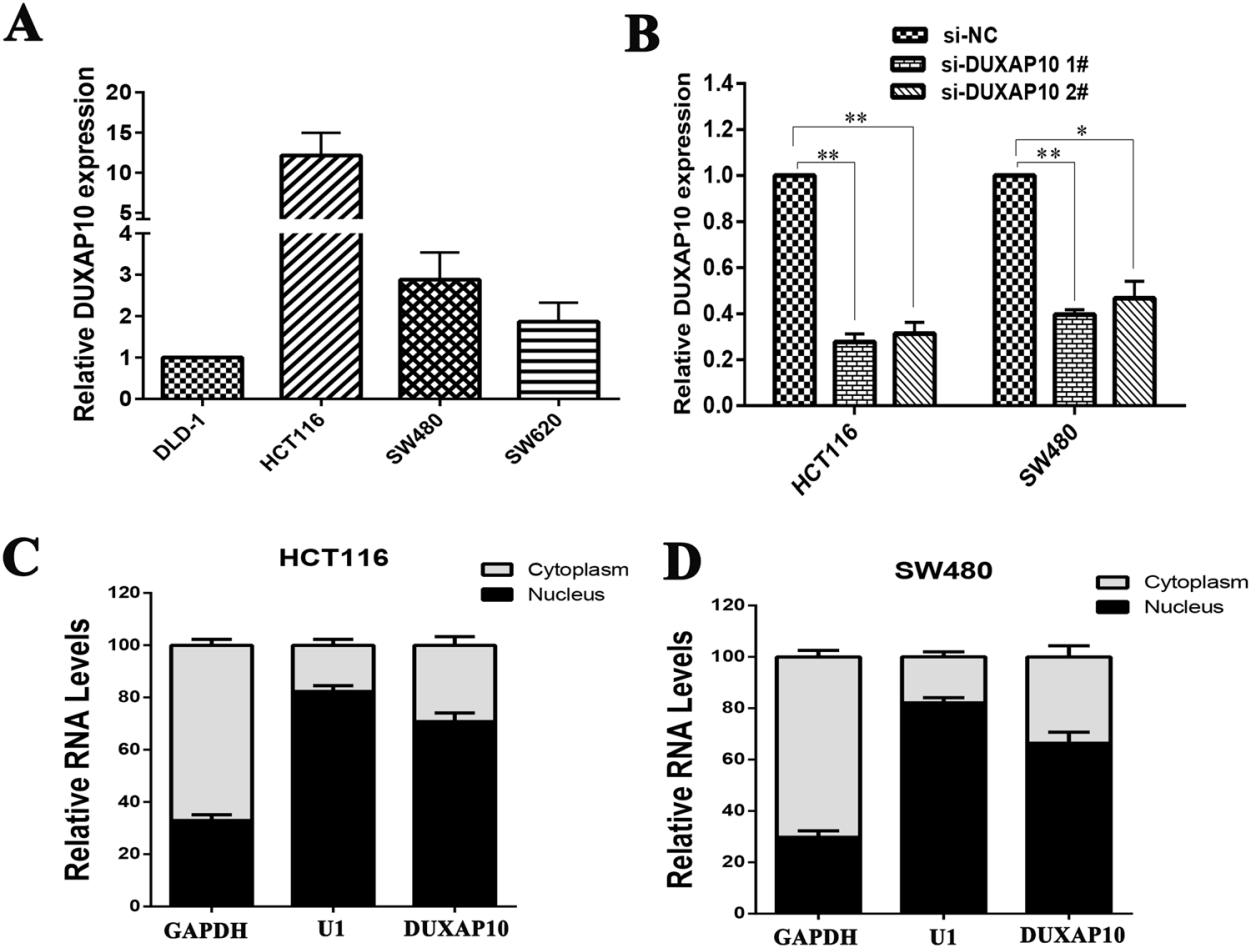
Relative DUXAP10 expression levels in CRC cell lines. (**A**) Analysis of DUXAP10 expression levels in CRC cell lines (DLD-1, HCT116, SW480 and SW620) by qPCR. **(B**) qPCR analysis of DUXAP10 expression levels following treatment of HCT116 and SW480 cells with siRNAs against DUXAP10. (**C**) Relative DUXAP10 levels in cell cytoplasm or nucleus of HCT116 and SW480 cell lines were detected by qPCR. Bars: s.d., *P < 0.05, **P < 0.01.

We next performed qPCR analysis to examine DUXAP10 expression in a panel of CRC cell lines, including DLD-1, HCT116, SW480 and SW620. We found that DUXAP10 expression levels were higher in HCT116 (*P* < 0.01), SW480 (*P* < 0.01) and SW620 (*P* < 0.05) cells than in DLD-1 cells (Fig. 2A). Then, to investigate the functional effects of DUXAP10 dysregulation in CRC cells, we knockdown endogenous DUXAP10 expression in HCT116 and SW480 cells by short interfering RNAs (siRNAs). qPCR analysis of DUXAP10 levels was performed 48 h post-transfection. The results showed that DUXAP10 expression was significantly reduced by si-DUXAP10 transfection compared with control cells (Fig. 2B). Next, to detect the distribution of DUXAP10 in colorectal cancer cells, we fractionated CRC cell lines into nuclear and cytoplasmic fractions, thoroughly separaing the nucleus from the cytoplasm. We found that DUXAP10 RNA was mostly located in the nucleus rather than the cytosol (Fig. 2C,D), suggesting that it may exert regulatory functions at the transcriptional level.

### Knockdown of DUXAP10 inhibits CRC cell proliferation, induces apoptosis, and promotes cell cycle arrest

**Figure 3:**
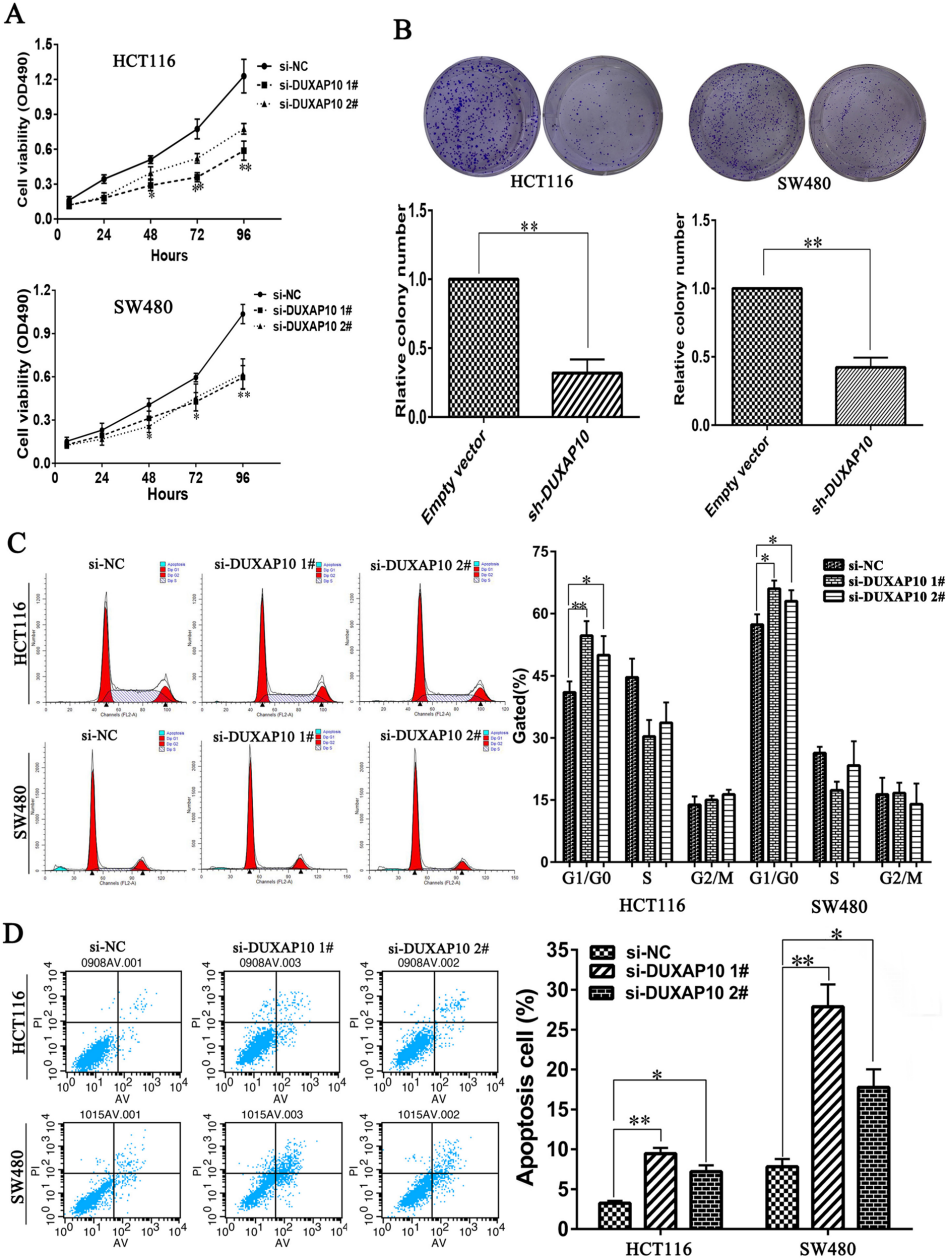
Effects of DUXAP10 on CRC proliferation and apoptosis *in vitro*. (**A**) MTT assays were performed to determine the cell viability of HCT116 and SW480 cells after the transfection of siRNA against DUXAP10. (**B**) Representative results of the colony formation of HCT116 and SW480 cells transfected with the siRNA of DUXAP10. (**C**) Flow cytometry assays were performed to analysis the cell cycle progression when HCT116 and SW480 cells transfected with siRNA against DUXAP10. (**D**) Flow cytometry assays were performed to analysis the cell apoptotic in siRNA-transfected HCT116 and SW480 cells. Representative images and data based on three independent experiments. Bars: s.d, *P < 0.05, **P < 0.01.

**Figure 4:**
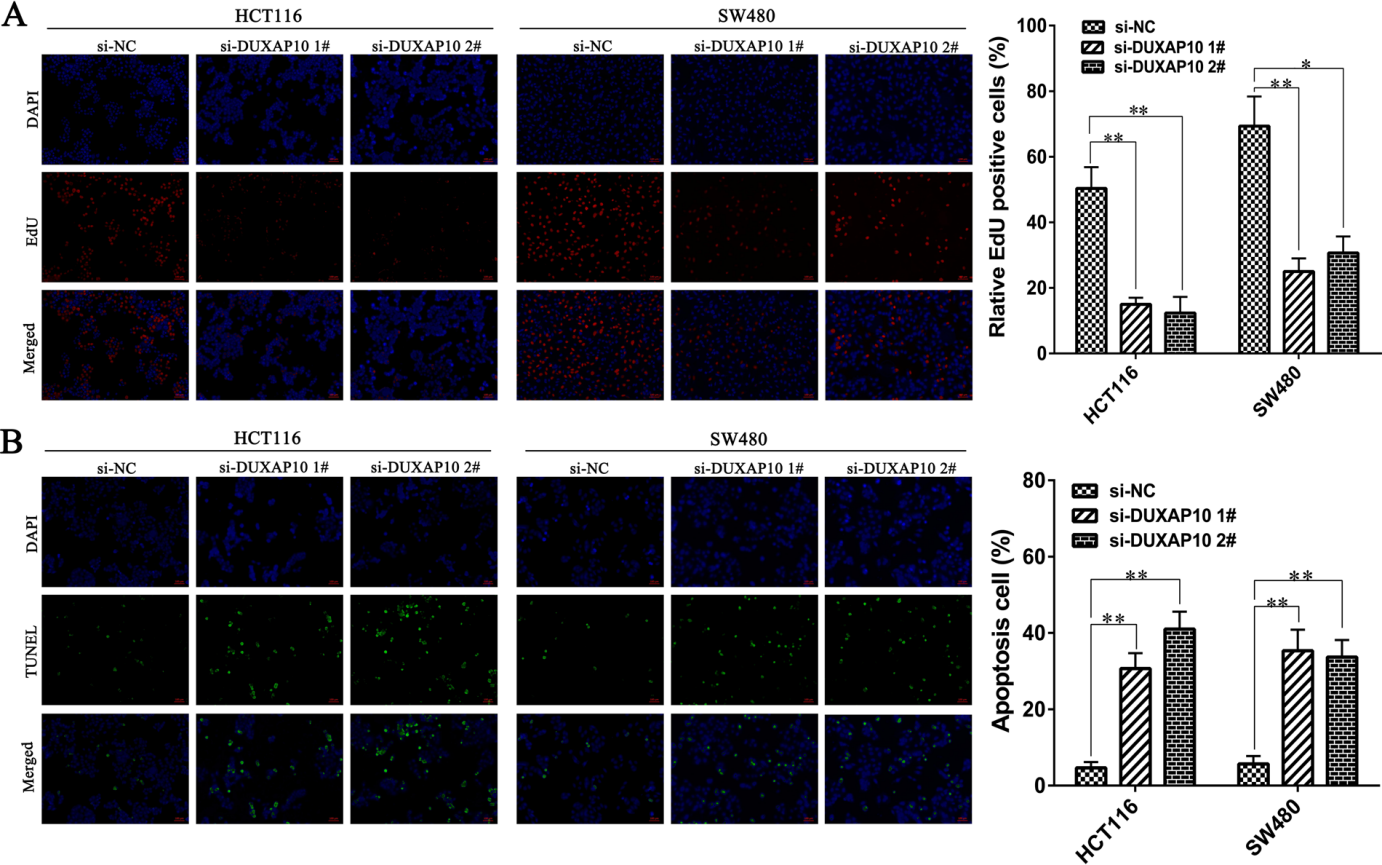
Effect of DUXAP10 on CRC cell proliferation and apoptosis confirmed by Edu analysis and TUNEL assay. (**A**) Proliferating HCT116 and SW480 cells were labeled with Edu. The Click-it reaction revealed Edu staining (red). Cell nuclei were stained with DAPI (blue). (**B**) TUNEL staining assays were performed to analyze cell apoptosis after DUXAP10 knockdown. The images of TUNEL positive cells were captured by a fluorescence microscope (200×). Quantitative result of TUNEL assay was analyzed. Representative images and data based on three independent experiments. Bars: s.d, *P < 0.05, **P < 0.01.

To further determine the biological role of DUXAP10 in CRC, we performed an MTT assay. The results showed that DUXAP10 knockdown significantly inhibited cell viability both in HCT116 and SW480 cell lines compared with control cells (Fig. 3A). Similarly, the colony formation assay results showed that clonogenic survival was strikingly decreased following DUXAP10 inhibition both in HCT116 and SW480 cell lines (Fig. 3B). Next, to determine whether the effects of DUXAP10 on CRC cell proliferation are the result of DUXAP10-mediated changes in cell cycle progression, we performed flow cytometry assay using HCT116 and SW480 cells. The results of si-DUXAP10 or si-NC transfection for 48 h showed that DUXAP10 knockdown increased the percentage of cells in G0/G1 phase and decreased the percentage of cells in S and G2/M phase compared to control cells (Fig. 3C). EdU analysis yielded similar results (Fig. 4A). To further investigated whether the effect of DUXAP10 on colorectal cancer cells proliferation reflected cell apoptosis, we performed flow cytometry and Tunel staining assays. The results showed that HCT116 and SW480 cells transfected with DUXAP10 siRNA had higher apoptotic rate in comparison with control cells (Figs 3D and 4B). These data suggest that DUXAP10 could promote the proliferation phenotype and inhibit apoptosis of colorectal cancer cells.

### DUXAP10 inhibits p21 and PTEN expression by binding to LSD1

**Figure 5:**
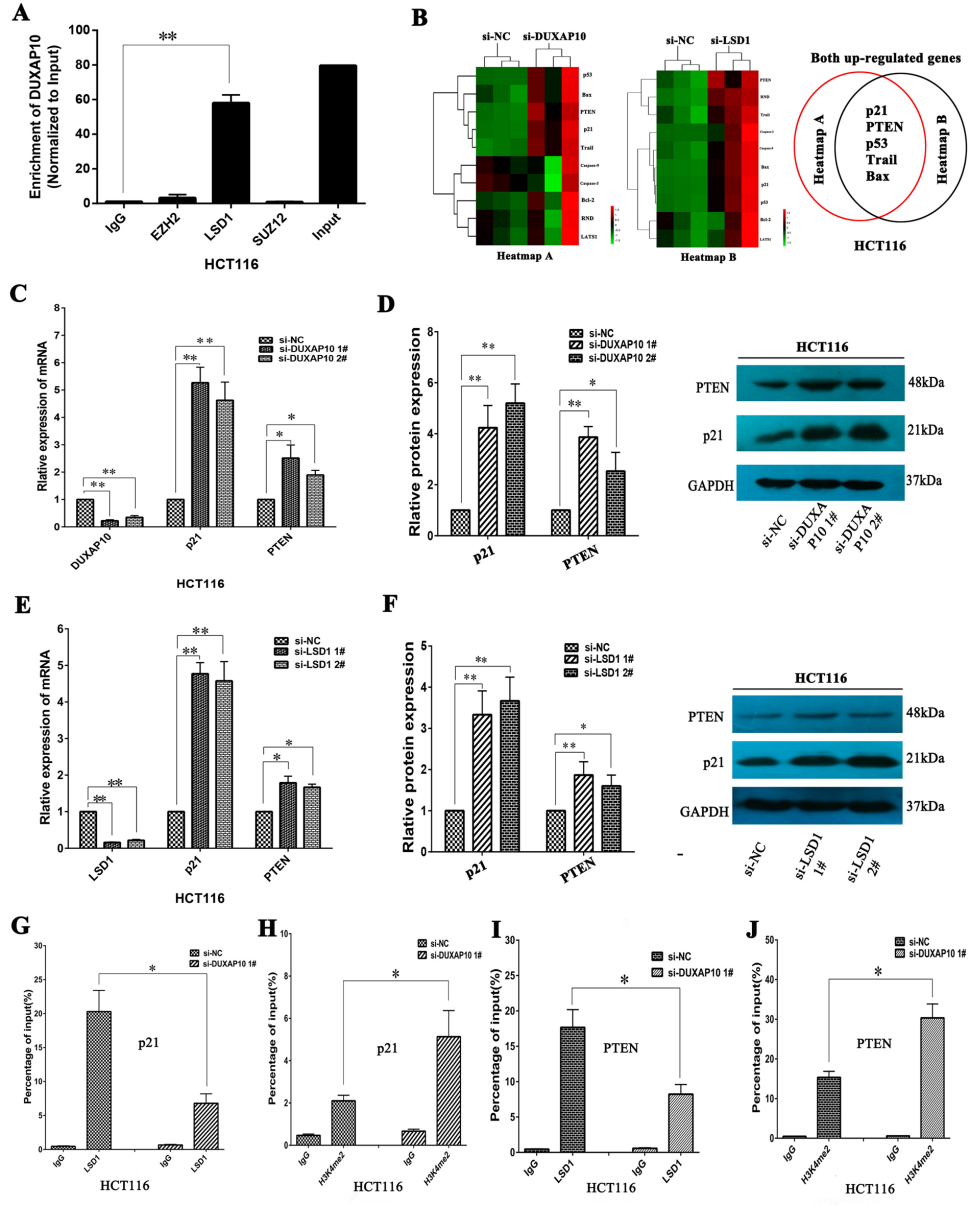
DUXAP10 epigenetically silences p21 and PTEN transcription by binding with LSD1. (**A**) RIP assays were performed in HCT116 cells and the coprecipitated RNA was subjected to qPCR for DUXAP10. (**B**) Heat maps of altered genes in DUXAP10 or LSD1 knockdown HCT116 cells compared with control cells. (**C**) The levels of p21 and PTEN mRNA were detected by qPCR when knockdown of DUXAP10 in HCT116 cells. (**D**) The p21 and PTEN protein levels were determined by western blot in DUXAP10 knockdown HCT116 cells. **(E,F)** The expression of p21 and PTEN in HCT116 cells, after knockdown of LSD1. **(G–J)** ChIP-qPCR of H3K4me2 and LSD1 of the promoter region of the p21 and PTEN locus after siRNA treatment targeting si-NC or si-DUXAP10 in HCT116 cells. Representative images and data based on three independent experiments. Bars: s.d, *P < 0.05, **P < 0.01.

Generally, lncRNAs regulate their target genes expression through interacting with RNA binding proteins such as polycomb repressive complex 2 (PRC2) or acting as endogenous competing RNAs (ceRNAs) for miRNAs *et al*. To investigate the molecular mechanism of DUXAP10 involved in colorectal cancer cells, we firstly predicted the interaction probabilities of DUXAP10 and RNA binding proteins via RNA-protein interaction prediction (http://pridb.gdcb.iastate.edu/RPISeq/), and found that DUXAP10 potentially binds EZH2, LSD1, and SUZ12 (as the RF or SVM score > 0.5). We next performed RIP assays and confirmed that DUXAP10 could interact with lysine-specific demethylase 1 (LSD1) but not other RNA binding proteins in HCT116 cells (Fig. 5A). Two Heat maps showed expression levels of 10 different cell growth related transcripts in HCT116 cells with knockdown of DUXAP10 or LSD1 expression for 48 hours (Fig. 5B). Analysis of these data, we found that p21, PTEN, p53, Trail and Bax were consistently up-regulated both in heat map A and heat map B (Fig. 5B). To prioritize the most DUXAP10 relevant genes, we focused on p21 and PTEN which are most highly expressed with knockdown of DUXAP10 or LSD1. Importantly, qPCR results also showed that inhibition of DUXAP10 or LSD1 expression led to increase p21 and PTEN expression in HCT116 cells (Fig. 5C,E). Furthermore, the western blot assays also showed the same results (Figs 5D,F), which indicated that p21 and PTEN might be DUXAP10 novel targets in CRC cells. To further determine whether DUXAP10 suppressed p21 and PTEN expression through interacting with LSD1, we performed chromatin immunoprecipitation analysis. The results showed that LSD1 could directly bind to p21 and PTEN promoter region and mediate H3K4me2 demethylation modification. However, knockdown of DUXAP10 reduced their binding ability and H3K4me2 demethylation modification (Fig. 5G–J). These data indicated that DUXAP10 contributes to CRC cell proliferation and apoptosis partly through repressing p21 and PTEN expression in CRC cells.

### Knockdown of DUXAP10 inhibits CRC cell tumorigenesis *in vivo*

**Figure 6:**
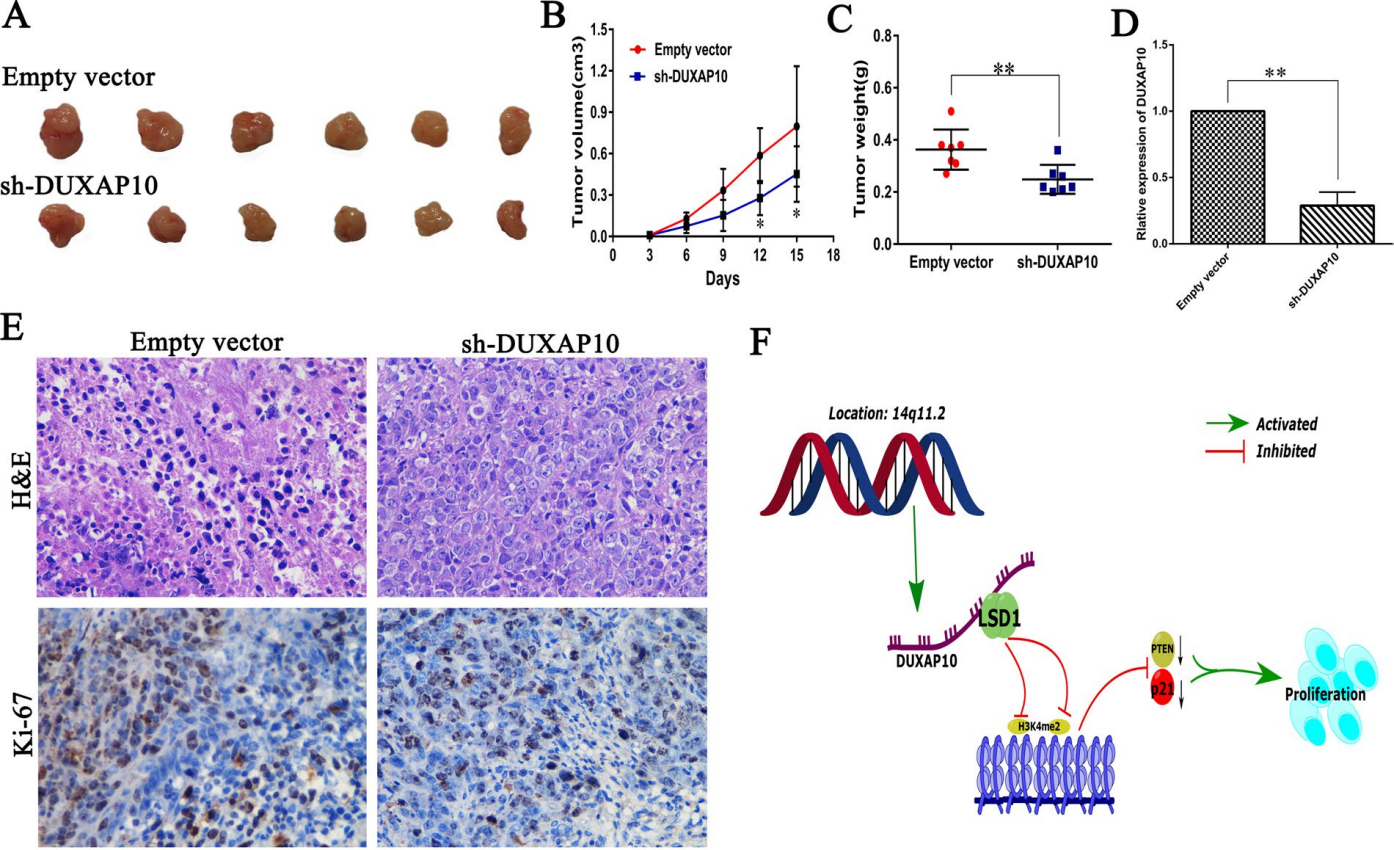
The silencing of DUXAP10 inhibited CRC growth *in vivo*. (**A**) The stable DUXAP10 knockdown HCT116 cells were used for the *in vivo* study. The nude mice carrying tumors from respective groups were shown. (**B**) Tumor volumes were calculated after injection every 3 days. **(C)** Tumor weights from two groups are represented. (**D**) qPCR was performed to detect the average expression of DUXAP10 in xenograft tumors (n = 6). (**E**) Images of HE staining and immunohistochemistry of the xenograft tumors. Representative Ki-67 protein levels in xenograft tumors as evaluated by IHC. (**F**) Summary diagram describes that DUXAP10 regulates CRC cell proliferation. Representative images and data based on three independent experiments. Bars: s.d, *P < 0.05, **P < 0.01.

Finally, to confirm the impact of DUXAP10 on CRC cell growth *in vivo*, HCT116 cells transfected with sh-DUXAP10 or empty vector were injected into male nude mice. The cells were transfected with empty vector as a control. At 15 days post-injection, the tumor growth in the sh-DUXAP10 group was markedly smaller than that in the control group (Fig. 6A). Correspondingly, the tumor volumes and weights were obviously decreased compared with the controls (Fig. 6B,C). As shown, qPCR confirmed that the DUXAP10 expression level was lower in the tumor tissues derived from the sh-DUXAP10-transfected cells (Fig. 6D). Moreover, immunohistochemistry (IHC) analysis confirmed that the tumors formed from HCT116/sh-DUXAP10 cells displayed weaker Ki-67 staining than those formed from the control cells (Fig. 6E). Our results indicated that silencing DUXAP10 expression could suppress colorectal cancer cells tumor growth *in vivo*.

## Discussion

The pseudogene was first reported by Jacq and colleagues in 1977^16^. Pseudogenes were originally considered mainly as ‘junk DNA,’ it was assumed that these transcripts contain no protein-coding capacity^17, 18^. Recently, evidence suggested that many pseudogene transcripts belongs to lncRNAs and involved in various biological process, including in cellular deregulation and the progression to cancer^19, 20^. However, to date, very few pseudogenes have been well characterized. Previous studies found that DUXAP10 was upregulated in non small cell lung cancer (NSCLC) and could promote cancer cell proliferation and invasion. They also found that RRAD and LATS2 were two direct target of DUXAP10^15^. Although DUXAP10 has been studied in NSCLC, the possible role of DUXAP10 in CRC remains to be clarified. In the present study, we revealed that pseudogene derived lncRNA DUXAP10 is upregulated in colorectal cancer tissues and DUXAP10 expression was significantly higher in patients with a larger tumor size, a higher pathological stage and lymph node metastasis. Further experiments demonstrated that knockdown of DUXAP10 induced cell apoptosis and inhibited cell proliferation in both HCT116 and SW480 cells. In contrast, DUXAP10 overexpression promoted the growth of CRC cells(Supplementary S2). Our findings showed that DUXAP10 may function as an oncogenic lncRNA in CRC and potentially be considered as a novel prognostic indicator for this disease.

A growing body of evidences suggest that lncRNA (including pseudogene RNAs) can upregulate target gene expression through binding RNA-binding proteins (RBPs) or competing for binding common miRNAs^21–27^. For example, lncRNA HOXA11-AS could promote proliferation and invasion of gastric cancer by scaffolding the chromatin modification factors PRC2, LSD1 and DNMT1^28^. The histone demethylase LSD1 plays an important role in the epigenetic regulation of gene transcription. More importantly, LSD1 is associated in many pathological processes of malignancy, such as carcinogenesis, proliferation, apoptosis and metastasis^29, 30^. A previous study demonstrated that by removing dimethylation of lysine 4 on histone H3 (H3K4m2) at the CDH-1 promoter, LSD1 downregulates the CDH-1 expression, and consequently promotes metastasis of colon cancer cells^31^. Interestingly, in this study, through RIP assays, ChIP assays and qPCR assays, we found that DUXAP10 can directly interact with LSD1 complexes and repress p21 and PTEN in HCT116 cells. Additionally, our results showed that LSD1 silenced p21 and PTEN expression by epigenetic regulation. While in SW480 cell lines, we founded that DUXAP10 could directly binds with EZH2 and does not bind with LSD1(Supplementary S1). Furthermore, ChIP analysis demonstrated that EZH2 directly binds to p21 promoter regions and induces H3K27me3 modification in SW480 cell lines(Supplementary S3). The inconsistency of DUXAP10 in regulating the target genes in HCT116 and SW480 cell lines may be due to its differential expression phenotype.

The functional role of p21 and PTEN has been previously illustrated in colorectal cancer^32, 33^. Numerous findings have shown that p21 and PTEN can function as tumor suppressors and closely related with cell proliferation and apoptosis^34^. We demonstrated that DUXAP10 which is associated with the RNA binding protein LSD1 to inhibit p21 and PTEN expression plays a critical role in the proliferation and tumorigenicity of colorectal cancer cells. However, other possible target genes and mechanism that underlie regulatory behaviors were not investigated in this study, which still remains to be fully understood and needs to be further investigated.

In conclusion, this study provides the first evidence that DUXAP10 is upregulated in CRC tissues and its overexpression may be associated with the poor prognosis of CRC patients. DUXAP10 can promote CRC cell proliferation and tumorigenesis partly via epigenetically silencing p21 and PTEN transcription by binding to LSD1 and preventing LSD1-mediated the demethylation modification of target genes. The oncogenic activity of DUXAP10 may serve as a novel biomarker and therapeutic target for CRC in future cancer clinic.

## Materials and Methods

### Tissue collection and ethics statement

A total of 58 patients analyzed in this study underwent resection of the primary colorectal cancer at the Second Affiliated Hospital of Nanjing Medical University. All collected tissue samples were immediately snap frozen in liquid nitrogen and stored at −80 °C until required. The study was approved by the Research Ethics Committee of Nanjing Medical University (Nanjing, Jiangsu, PR China), and written informed consent was obtained from all patients. The clinicopathological characteristics of the colorectal cancer patients are summarized in Table 1. We confirm that all methods were performed in accordance with the relevant guidelines and regulations.

### Total RNA isolation and qPCR assays

Total RNA was extracted from tissues or cultured cells using TRIzol reagent (Invitrogen, Carlsbad, CA) according to the manufacturer’s protocol. RNA quantity and quality were determined by NanoDrop2000c (Thermo Scientific, Waltham, MA, USA). For qPCR, 1 μg of RNA was reverse transcribed to cDNA using a Reverse Transcription Kit (Takara, Dalian, China). The quantitative polymerase chain reaction (qPCR) assays were conducted on an ABI 7500. Data were normalized to the expression of glyceralde-hyde-3-phosphatedehydrogenase (GAPDH). Primers used for target amplification are listed in Supplementary Table S1.

### Cell lines and culture conditions

Four CRC cell lines (DLD-1, HCT116, SW480 and SW620) were obtained from the American Type Culture Collection (Manassas, VA). All of the cell lines were grown and maintained in RPMI 1640 Medium (Invitrogen) and supplemented with 10% fetal bovine serum (FBS), 100 U/ml penicillin, and 100 mg/ml streptomycin (Invitrogen, Shanghai, China) at 37 °C with 5% CO_2_.

### Cell transfection

Typically, CRC cells were seeded at six-well plates and then transfected in the next day with specific siRNA (100 nM) or control siRNA (100 nM) using Lipofectamine 2000 (Invitrogen), according to the manufacturer’s protocol (Invitrogen). After transfection, the cells were harvested for further studies. The primer sequences and siRNA sequences are summarized in Supplementary Table S1.

### Subcellular fractionation location

The separation of nuclear and cytosolic fractions was performed using the PARIS Kit (Life Technologies) according to the manufacturer’s instructions.

### Cell viability and colony formation assay

Cell viability was monitored using the Cell Proliferation Reagent Kit I (MTT; Roche Applied Science). The HCT116 and SW480 cells were transfected with siRNA or si-NC (3000 cells/well) and were cultured in 96-well plates with six replicate wells. Cell viability was assessed according to the manufacturer’s recommendations. For the colony formation assay, a total of 500 cells were placed in a six-well plate and maintained in media containing 10% FBS. The medium was replaced every 4 days. After 2 weeks, cells were fixed with methanol and stained with 0.1% crystal violet (SigmaAldrich). Visible colonies were manually counted. Triplicate wells were measured in each treatment group.

### Flow cytometry

HCT116 and SW480 cells transfected with siRNA or si-NC were harvested after 48 h. Subsequently, the cells were stained with PI using the CycleTESTTM PLUS DNA Reagent Kit (BD Biosciences) according to the protocol and analyzed with a flow cytometer (FACScan®;BDBiosciences) equipped with the CellQuest software (BD Biosciences). The percentages of the cells in G0-G1, S, and G2-M phases were calculated and compared.

HCT116 and SW480 cells transfected with siRNA or si-NC were harvested after 48 h for apoptosis analysis. The cells were then treated with fluorescein isothiocyanate (FITC) Annexin V and propidium iodide (PI) in the dark at room temperature according to the manufacturer’s recommendations. Subsequently, the cells were analyzed by FACScan®, and they were identified as viable, dead, early apoptotic, or late apoptotic cells.

### EdU analysis and TUNEL staining assay

EdU analysis and TUNEL staining assay were performed as previously reported in Lian *et al*.^35, 36^.

### *In vivo* tumor formation assay

All the animal experiments in this study were performed in strict accordance with the guidelines and regulations for the Care and use of Laboratory Animals, Nanjing Medical University, China. The protocols were approved by the Research Ethics Committee of Nanjing Medical University, China. Four-week-old male athymic mice were purchased from the Animal Center of the Nanjing University (Nanjing, China) and maintained in pathogen-free conditions. HCT116 cells were transfected with sh-DUXAP10 or empty vector and harvested from six-well plates, washed with phosphate-buffered saline (PBS), and resuspended at 2 × 10^7^ cells/mL. Subsequently, each mouse was injected into the lower right flank with 100 μL of suspended cells. Tumor growth was examined every 3 days, and tumor volumes were measured as the length × width^2^ × 0.5. At 15 days post-injection, mice were sacrificed by CO_2_ asphyxiation, and the growth of each tumor was examined.

### RNA immunoprecipitation (RIP)

RNA immunoprecipitation was used to investigate whether DUXAP10 could interact or bind with the potential binding protein (EZH2, SUZ12 and LSD1.) in HCT116 cells. We used the EZMagna RIP kit (Millipore, Billerica, MA, USA) following the manufacturer’s protocol. HCT116 cells were lysed in complete RIP lysis buffer, and the extract was incubated with magnetic beads conjugated with antibodies that recognized EZH2, SUZ12, LSD1 or control IgG (millipore) for 6hr at 4 °C. Then, the beads were washed and incubated with Proteinase K to remove proteins. Finally, purified RNA was subjected to qPCR analysis to demonstrate the presence of DUXAP10 using specific primers.

### Chromatin immunoprecipitation (ChIP)

HCT116 cells were treated with formaldehyde and incubated for 10 mins to generate DNA-protein cross-links. Cell lysates were then sonicated to generate chromatin fragments of 200–300 bp and immunoprecipitated with H3K4me2, LSD1 and IgG as control. Precipitated chromatin DNA was recovered and analyzed by qPCR. The primer sequences used for the studies are shown in Supplementary Table S1.

### Western blot assay

HCT116 cells were harvested, and protein was extracted from transfected cells and quantified as previously described using 12% polyacrylamide gradient SDS gel. Anti-GAPDH, anti-p21 and anti-PTEN were from Abcam (Hong Kong, China). ECL chromogenic substrate was used to were quantified by densitometry (Quantity One software; Bio-Rad). GAPDH antibody was used as control.

### Immunohistochemistry (IHC)

Xenograft tumor tissue samples were immunostained for H&E and Ki67. Anti-Ki67 was from Santa Cruz Biotechnology (Dallas, TX, USA). The IHC staining results were independently scored by the author and a pathologist to minimize subjectivity and then compared, and the final comprehensive results were obtained.

### Statistical analysis

All statistical analyses were performed using SPSS software, version 22.0 (SPSS, Chicago, IL, USA). A paired, two-tailed Student’s *t*-test or a chi-square test was used to evaluate significant differences between groups of data. All data are represented as means ± SD. Differences were considered significant if *P* < 0.05. “*” indicates *P* < 0.05; “**” indicates *P* < 0.01.

## Electronic supplementary material

Supplementary Figures-1

Supplementary Figures-2

Supplementary Table S1
