# Supplementary material for: The pseudogene derived from long non-coding RNA DUXAP10 promotes colorectal cancer cell growth through epigenetically silencing of p21 and PTEN
Source: Sci Rep. 2017 Aug 4;7:7312. doi: 10.1038/s41598-017-07954-7 (PMC5544748; doi:10.1038/s41598-017-07954-7)
Supplement: Supplementary file 1 — Supplementary Figures-1 [file 41598_2017_7954_MOESM1_ESM.pdf]

The pseudogene derived from long non-coding RNA DUXAP10 promotes colorectal cancer cell growth through epigenetically silencing of p21 and PTEN.

Yifan Lian[1,2,6], Yetao Xu[3,6], Chuanxing Xiao[1,6], Rui Xia[4], Huangbo Gong[5], Peng Yang[5], Tao Chen[5], Dongdong Wu[5], Zeling Cai[5], Jianping Zhang[5], Keming Wang[2*]

[1]Department of Gastroenterology, Zhongshan Hospital affiliated to Xiamen University, Xiamen, 361004, Fujian, People's Republic of China; [2]Department of Oncology, Second Affiliated Hospital, Nanjing Medical University, Nanjing 210000, Jiangsu, People's Republic of China; [3]Department of Obstetrics and Gynecology, the First Affiliated Hospital of Nanjing Medical University, Nanjing, 210000, Jiangsu, People's Republic of China; [4]Department of Laboratory, Nanjing Chest Hospital, Nanjing, 210029, Jiangsu, People's Republic of China; [5]Department of General Surgery, Second Affiliated Hospital, Nanjing Medical University, Nanjing,210000 Jiangsu, People's Republic of China.

[6]This authors contributed equally to the work.

[*]**Corresponding author:** Keming Wang, E-mail: kemingwang@njmu.edu.cn, Tel: +86-18951762692, Fax : +86-25-58509994

Supplementary Figures-1

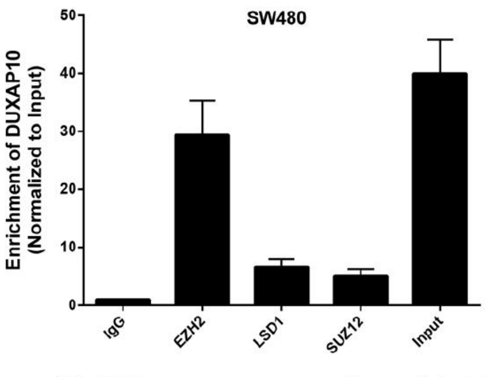

Supplementary Figure S1: RIP assays were performed in SW480 cells and confirmed that DUXAP10 could interact with EZH2 in SW480 cells.

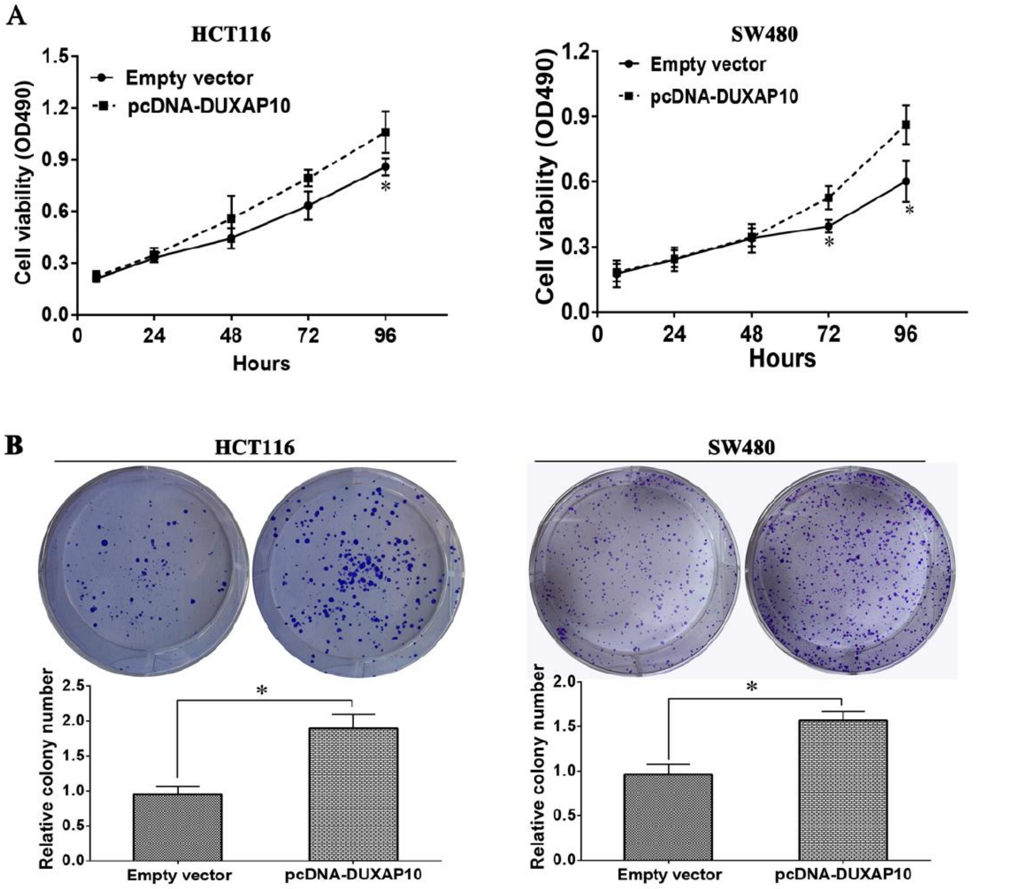

Supplementary Figure S2: Overexpression of DUXAP10 on CRC cell proliferation in vitro. (A and B). Cell viability and colony formation assay were used to determine the proliferation of pcDNA-DUXAP10-transfected HCT116 and SW480 cells.
