# Supplementary material for: The pseudogene derived from long non-coding RNA DUXAP10 promotes colorectal cancer cell growth through epigenetically silencing of p21 and PTEN
Source: Sci Rep. 2017 Aug 4;7:7312. doi: 10.1038/s41598-017-07954-7 (PMC5544748; doi:10.1038/s41598-017-07954-7)
Supplement: Supplementary file 2 — Supplementary Figures-2 [file 41598_2017_7954_MOESM2_ESM.pdf]

The pseudogene derived from long non-coding RNA DUXAP10 promotes colorectal cancer cell growth through epigenetically silencing of p21 and PTEN.

Yifan Lian[1,2,6], Yetao Xu[3,6], Chuanxing Xiao[1,6], Rui Xia[4], Huangbo Gong[5], Peng Yang[5], Tao Chen[5], Dongdong Wu[5], Zeling Cai[5], Jianping Zhang[5], Keming Wang[2*]

[1]Department of Gastroenterology, Zhongshan Hospital affiliated to Xiamen University, Xiamen, 361004, Fujian, People's Republic of China; [2]Department of Oncology, Second Affiliated Hospital, Nanjing Medical University, Nanjing 210000, Jiangsu, People's Republic of China; [3]Department of Obstetrics and Gynecology, the First Affiliated Hospital of Nanjing Medical University, Nanjing, 210000, Jiangsu, People's Republic of China; [4]Department of Laboratory, Nanjing Chest Hospital, Nanjing, 210029, Jiangsu, People's Republic of China; [5]Department of General Surgery, Second Affiliated Hospital, Nanjing Medical University, Nanjing,210000 Jiangsu, People's Republic of China.

[6]This authors contributed equally to the work.

[*]**Corresponding author:** Keming Wang, E-mail: kemingwang@njmu.edu.cn, Tel: +86-18951762692, Fax : +86-25-58509994

Supplementary Figures-2

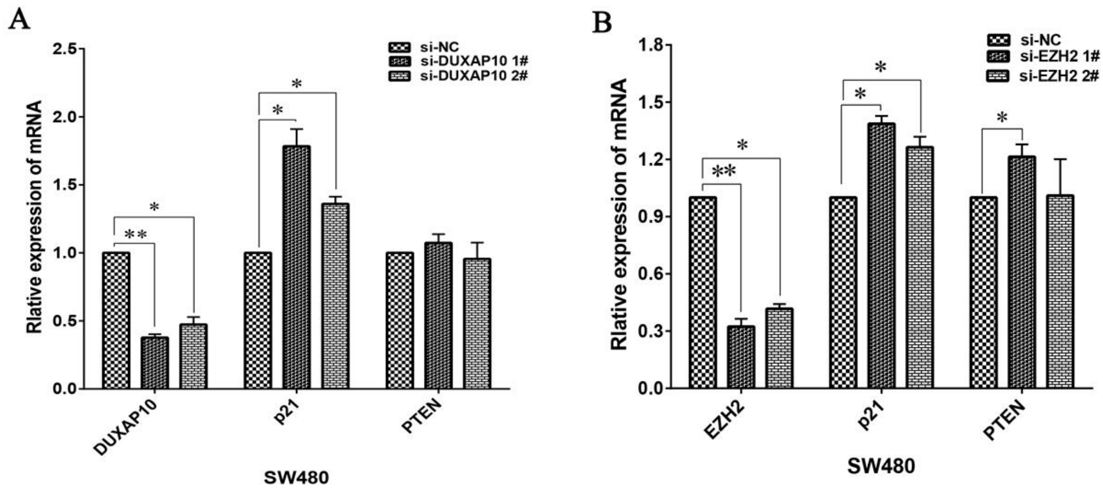

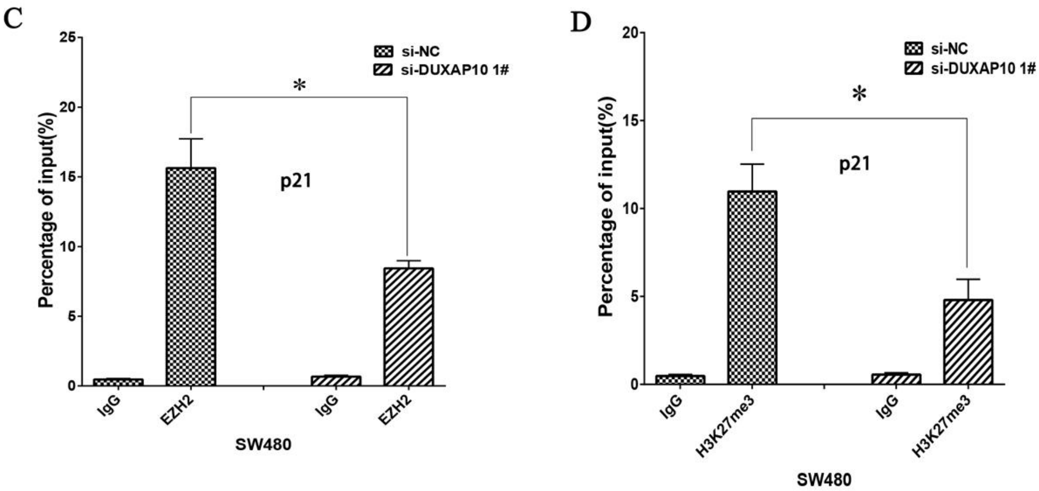

Supplementary Figure S3: (A) The levels of p21 and PTEN mRNA were detected by qPCR when knockdown of DUXAP10 in SW480 cells.(B) The expression of p21 and PTEN in SW480 cells, after knockdown of LSD1. (C and D) ChIP-qPCR of H3K27me3 and EZH2 of the promoter region of the p21 locus after siRNA treatment targeting si-NC or si-DUXAP10 in SW480 cells. Representative images and data based on three independent experiments. Bars: s.d, *P<0.05, **P<0.01.
