# Supplementary material for: The pseudogene derived from long non-coding RNA DUXAP10 promotes colorectal cancer cell growth through epigenetically silencing of p21 and PTEN
Source: Sci Rep. 2017 Aug 4;7:7312. doi: 10.1038/s41598-017-07954-7 (PMC5544748; doi:10.1038/s41598-017-07954-7)
Supplement: Supplementary file 3 — Supplementary Table S1 [file 41598_2017_7954_MOESM3_ESM.pdf]

The pseudogene derived from long non-coding RNA DUXAP10 promotes colorectal cancer cell growth through epigenetically silencing of p21 and PTEN.

Yifan Lian[1,2,6], Yetao Xu[3,6], Chuanxing Xiao[1,6], Rui Xia[4], Huangbo Gong[5], Peng Yang[5], Tao Chen[5], Dongdong Wu[5], Zeling Cai[5], Jianping Zhang[5], Keming Wang[2*]

[1]Department of Gastroenterology, Zhongshan Hospital affiliated to Xiamen University, Xiamen, 361004, Fujian, People's Republic of China; [2]Department of Oncology, Second Affiliated Hospital, Nanjing Medical University, Nanjing 210000, Jiangsu, People's Republic of China; [3]Department of Obstetrics and Gynecology, the First Affiliated Hospital of Nanjing Medical University, Nanjing, 210000, Jiangsu, People's Republic of China; [4]Department of Laboratory, Nanjing Chest Hospital, Nanjing, 210029, Jiangsu, People's Republic of China; [5]Department of General Surgery, Second Affiliated Hospital, Nanjing Medical University, Nanjing,210000 Jiangsu, People's Republic of China.

[6]This authors contributed equally to the work.

[*]**Corresponding author:** Keming Wang, E-mail: kemingwang@njmu.edu.cn, Tel: +86-18951762692, Fax : +86-25-58509994

Supplementary Table S1

Primers used for qPCR	Sequences (5' to 3')
DUXAP10 (Forward)	CTGTAGGAGGCCAAGACAGG
DUXAP10 (Reverse)	CATTGTCTCAAGGTCTGCTGAA
GAPDH (Forward)	GAAGAGAGAGACCCTCACGCTG
GAPDH (Reverse)	ACTGTGAGGAGGGGAGATTCAGT
SUZ12 (Forward)	TGCAGTTCACTCTTCGTTGG
SUZ12 (Reverse)	TGCTTCAGTTTGTTGCCTTG
LSD1 (Forward)	AGCGTCATGGTCTTATCAA
LSD1 (Reverse)	GAAATGTGGCAACTCGTC
EZH2 (Forward)	TGCACATCCTGACTTCTGTG
EZH2 (Reverse)	AAGGGCATTCACCAACTCC
P21 (Forward)	AAGTCAGTTCCTTGTGGAGCC
P21 (Reverse)	GGTTCTGACGGACATCCCCA
PTEN (Forward)	TGGATTCGACTTAGACTTGACCT
PTEN (Reverse)	GGTGGGTTATGGTCTTCAAAAGG
U1 (Forward)	GGGAGATACCATGATCACGAAGGT
U1 (Reverse)	CCACAAATTATGCAGTCGAGTTTCCC
Primer used for qCHIP analysis	Sequences (5' to 3')
PTEN(Forward)	CCGAATCAGCTCTCTCACGG
PTEN5(Reverse)	GCGTGCATTCTCCGATGTTG

P21 (Forward)	GCCTTCCTCACATCCTCC
P21 (Reverse)	CAAGAGTGCCCAGTCCAG
Interference sequences (siRNA)	
siRNA 1# (DUXAP10)	GGAACUUCCCAAACCUCCAUGAUUU
siRNA 2# (DUXAP10)	CAGCAUACUUCAAAUUCACAGCAAA
siRNA 1# (LSD1)	CAUUUGAGGCUACUCUCCAACAAUU
siRNA 2# (LSD1)	CAAAGGAUGGGAUUUGGCAACCGGA
si-NC	UUCUCCGAACGUGUCACGUTT
